# Heterogenic Solid Biofuel Sampling Methodology and Uncertainty Associated with Prompt Analysis

**DOI:** 10.3390/ijms11052118

**Published:** 2010-05-11

**Authors:** Jose A. Pazó, Enrique Granada, Ángeles Saavedra, David Patiño, Joaquín Collazo

**Affiliations:** 1 ETS Ingenieros Industriales, University of Vigo, Lagoas-Marcosende s/n 36200-Vigo, Spain; E-Mails: jpazo@uvigo.es (J.A.P.); patinho@uvigo.es (D.P.); joaquincollazo@uvigo.es (J.C.); 2 ETS Ingeniería de Minas, University of Vigo, C.P. 36310 Vigo (Pontevedra), Spain; E-Mail: saavedra@uvigo.es

**Keywords:** solid biofuel, sampling methodology, uncertainty, prompt analysis

## Abstract

Accurate determination of the properties of biomass is of particular interest in studies on biomass combustion or cofiring. The aim of this paper is to develop a methodology for prompt analysis of heterogeneous solid fuels with an acceptable degree of accuracy. Special care must be taken with the sampling procedure to achieve an acceptable degree of error and low statistical uncertainty. A sampling and error determination methodology for prompt analysis is presented and validated. Two approaches for the propagation of errors are also given and some comparisons are made in order to determine which may be better in this context. Results show in general low, acceptable levels of uncertainty, demonstrating that the samples obtained in the process are representative of the overall fuel composition.

## 1. Introduction

Global concern about environmental protection has grown considerably in the last few decades, culminating in the Kyoto Protocol [1], which set major directives and acceptable pollutant emission levels. The 2009 United Nations Climate Change Conference has recognized that climate change is one of our main challenges and some actions should be taken to avoid any temperature increase. The Copenhagen Summit ratified the Kyoto protocol, which expires in 2012, to continue its work but there was no agreement in relation to emissions reduction beyond 2012. The final agreement has no legal binding. After Kyoto, considerable efforts have been made to measure and control pollutant output from all energy processes and especially to minimize greenhouse gases. Developing renewable, clean energies such as biomass has become an important working area as part of the action plan drawn up. Biomass cofiring has in fact become one of the most profitable ways of reducing pollutant emissions from energy production because the adjustments required to power plants entail low costs [2–4].

Several different technologies are normally applied in cofiring processes [5]. The main advantages of each have been highlighted by various authors [2–9]. In short, cofiring can be said to help reduce specific emissions of CO_2_ due to the closed carbon cycle; the low sulfur content of biomass helps minimize SO_2_ emissions, and NO_x_ also shows a positive trend. Cofiring increases the operational flexibility of the process, reducing dependence on fossil fuels such as coal, but its main drawbacks are the additional cost of adapting combustion facilities and the increase in fouling and corrosion of equipment [5].

**Figure 1.**
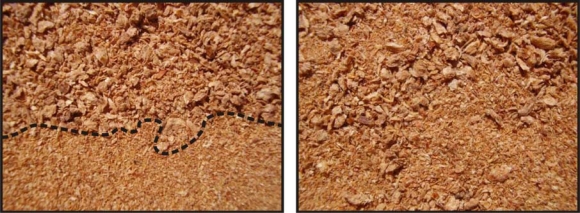
Different segregation states for the same sample. The picture on the left shows a high degree of segregation while the one on the right shows the opposite case.

To avoid some of these problems, it is important properly to define the composition of the biomass used for cofiring. This is made more difficult by the high intrinsic heterogeneity of solid biofuels, so a well-defined measurement methodology must be developed to ensure declared characteristics with an acceptable, clearly defined level of uncertainty. Many reference studies have been published dealing with this issue [10–14] and proposing various sampling methods. Methodologies are often based on small samples from large batches which require careful reduction to avoid segregation and stratification problems [13] as shown in Figure 1. A good sampling method should be able to get a representative sample with no influence from these problems.

The present paper presents a new methodology for solid biomass fuel sampling and error determination independently of the origin, appearance and packaging of the batch. To validate this procedure, prompt analysis of different biofuels is carried out. Moisture, volatile matter and ash content are obtained directly from a series of samplings and fixed carbon content is inferred from them. Moisture content influences the low heating value, ash is critical in the effects of fouling and corrosion [15,16] and volatiles characterize the behaviour of the flame. The overall uncertainty of the measurements is defined, allowing us to determine the minimum number of samples needed to achieve an acceptable level of reliability.

Since fixed carbon content can be calculated as a function of moisture, volatile matter and ash content, the uncertainties of these last three properties propagate the uncertainty of fixed carbon. In this paper a new approach for approximating error propagation is derived. This expression is compared to the traditional formula that can be seen in [17]. The results, presented in Section 3, show a substantial improvement in the approximation of the error.

## 2. Experimental Methodology

All materials manipulations were developed in the same laboratory and by the same analyst. As the materials exposure after sampling to environmental conditions are less than half an hour in the worst case, we ignore the effects of environmental variations in the material properties (temperature and relative humidity variations in the laboratory are considered insignificant in such a short period of time). Laboratory instruments have been verified and calibrated in order to assure the accuracy of the experimental methodology. Errors registered during the realization of the experiments are considered to be non-systematic errors and therefore related to the precision of the experiment. These latest errors are quantified in the total sampling error.

### 2.1. Materials

Several different materials from agriculture and forestry were selected for the study, covering a broad spectrum of solid biomass which could be used as fuel in cofiring processes. The agricultural materials were stored in big-bags and the forestry materials, in pellet form, were stored in sacks. The materials of agricultural origin selected were pine kernel shells, almond shells, hazelnut shells and ground olive stones. The materials of forest origin were pine pellets, oak pellets, brassica pellets and poplar pellets.

### 2.2. Sampling

Depending on the material, sampled masses vary from 320 × 10^−3^ kg to 730 × 10^−3^ kg. Fuel samples were obtained through a tube sampler, which was designed to work with all kinds of solid biomass. In its construction special attention was paid to the fact that biofuels are supplied in sacks or big-bag. The nominal maximum size “d” of the material sampled is taken as 20 mm [18], so the tube sampler should be able to collect at least than V_min_ = 0.05 × d = 0.05 × 20 = 1 dm^3^ = 10^−3^ m^3^ [19].

**Figure 2.**
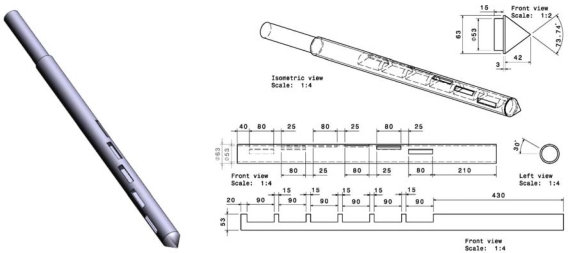
3D illustration and technical drawing of the tube sampler.

The tube sampler comprises three parts (Figure 2). The first part is the outer tube, which has six rectangular holes placed 30 degrees apart. The holes measure 80 × 30 mm, with the longest dimension in the direction of the axis. The second piece is the inner tube, which can be rotated within the outer tube so that the holes can be closed while the tube is inserted into the sample and then opened when the device is in the correct position for collecting the sample. The third piece is fitted to the tip of the outer tube to help the device penetrate the sack of material under study. The instrument is easy to clean thanks to the removable cone. The design is based on the standard [19] and the work of Pierre Gy [20].

#### 2.2.1. Big-Bag Procedure

The different biomasses contained in big-bags (1.5 m^3^ approximately) were Hazelnut shell, Pine nut shell, Almond shell and ground Olive stone, each biomass in its own big-bag. Nine samples of approximately 10^−3^ m^3^ volume were extracted [19]. The upper surface of the big-bag, which is circular, was divided into eight equal circles. Samples were removed from each circular sector by inserting a tube sampler at 2/3 from the centre. The ninth sample was removed from the centre of the big-bag.

#### 2.2.2. Pellet Sack Procedure

The different biomasses contained in sacks (0.025 m^3^ approximately) were poplar pellets (nine sacks), brassica pellets (25 sacks), oak pellets (10 sacks) and pine pellets (24 sacks). Samples of about 10^−3^ m^3^ volume were collected from 5 selected sacks using a table of random numbers [19]. Samples were obtained by first inserting the tube sampler from one top corner of the sack to the opposite bottom corner and then repeating the process from the opposite corner. The two samples from each bag were mixed and stored in the same bottle, thereby obtaining five bottles of each sample material. In the case of pine and oak pellets the process was analogous but samples from the same bag were not mixed, so ten sample bottles were obtained. The bottles used to store the samples were made of polypropylene, wide-necked with a lid and screw top and therefore air-tight.

### 2.3. Reduction of the Samples

The samples that were laboratory tested had to be reduced in size; the process was the same for all samples:

1. The selected samples were completely ground in a RETSCH SM-100 grinder, using a 6 mm nominal square step sieve. This filter was chosen because this particle size is large enough to be used even for cofiring with coal [21]. The olive stone samples did not receive this treatment because they were delivered already ground to a smaller size. After grinding, samples were stored back in the original bottles.
2. The sample was divided into similar parts using a slotted box called a Boerner divider, which separated them into smaller samples. Table 1 shows the rounded-off average weights of the samples selected for analysis of each material. Once a sample was selected, it was separated into two halves. One part was tested to determine the moisture content and the other was stored.
3. The moisture content was determined. Dry samples were stored in new bags from which the sample for the ash test was obtained. Before testing, the sample was ground in a mill with IKA MF 10.2, with an impact grinding head, producing particle sizes of less than 3 × 10^−3^ m, to determine the ash content.
4. The dry samples obtained in the previous step were divided into two parts, one of which was used to determine the volatile matter content and the other to determine the ash content, except for hazelnut shell, oak and pine pellets samples, which were studied wet.

### 2.4. Test Methodology

#### 2.4.1. Moisture Content

The method used was oven drying (Nabertherm) of the wet sample obtained by the reduction procedure described above. Aluminium trays with an interior diameter of 0.093 m which were free from corrosion and had no moisture adsorption were used.

The samples were weighed using the “Great Series VXI-110” scale, which is accurate to 10^−8^ kg. The empty tray was weighed, then the sample was uniformly distributed over the surface of the tray with less than 10^−3^ kg/10^−4^ m^2^. The weighed samples of each material were simultaneously placed in the furnace at a temperature of 105 °C. The time spent on stabilizing these conditions was 180 minutes, to ensure constant mass. Moisture content when wet (M_i)_ was obtained by the following equation [22]:

(1) $${\text{M}}_{\text{i}}=\frac{{(\text{m}}_{2}-{\text{m}}_{3})-({\text{m}}_{4}-{\text{m}}_{5})+{\text{m}}_{6}}{\left({\text{m}}_{2}-{\text{m}}_{1}\right)}\times 100$$

where the different m_i_ (10^−3^ kg) indicate:

m_1_: Empty tray
m_2_: Tray and sample before drying
m_3_: Tray and sample after drying
m_4_: Reference tray at room temperature before drying
m_5_: Tray after drying when reference is still hot
m_6_: Moisture of the packing if necessary

#### 2.4.2. Ash Content

The ash is the residual inorganic mass which remained after combustion of a biofuel sample at a controlled temperature of 550 ± 10 °C in oven air until constant mass was established [23]. To set up the tests, SiO_2_ and Al_2_O_3_ crucibles were used as recipients. Their properties include chemical stability, low mechanical strength expansion at high temperature and thermal shock resistance [24]. The sample covered the surface of the container in a proportion of no more than, 10^−4^ kg/10^−4^ m^2^, the smallest amount tested was 10^−3^ kg. To weigh the samples, scales accurate to 10^−8^ kg were again used. The sample was ground and passed through the 3 MF 3 mm sieve. Before the tests, the crucibles were placed in the oven at 550 ± 10 °C for 60 min. The sample was placed in the crucible and uniformly distributed over the bottom surface. The dry sample and crucible were weighed and then put into the oven when cold in order to start the test. A heating rate of 5 °C/min to 250 °C was programmed. Once finished, the temperature was kept at 250 °C for 60 min to evaporate the volatiles. With the same heating rate, the temperature was increased to 550 ± 10 °C and held for 360 min. The ash content when dry, Ai, was calculated by [23].

(2) $${\text{A}}_{\text{i}}=\frac{\left({\text{m}}_{3}-{\text{m}}_{1}\right)}{{(\text{m}}_{2}-{\text{m}}_{1})}\times 100$$

where the different m_i_ (10^−3^ kg) indicate:

m_1_: empty crucible.
m_2_: crucible and sample.
m_3_: crucible and ash.

#### 2.4.3. Volatile Content

The volatile matter content was determined using a special furnace (CARBOLITE ELF 11/68) with a maximum temperature of 1100 °C [25]. The sample was placed in covered crucibles at a temperature of 900 °C for 7 minutes. After that, the crucibles were removed and cooled for 10 minutes at room temperature and then placed in a dryer to bring them into thermal equilibrium with the room. This methodology is based on the procedure described in [26].

Crucible tips fitted perfectly and the sample was uniformly distributed over their inner surfaces. Volatile matter content was determined by weight difference, as shown in equation 3. The scale used is accurate to 10^−8^ kg

(3) $$V_{i}=\frac{\left(m_{2}-m_{3}\right)}{\left(m_{2}-m_{1}\right)}\times 100$$

where:

m_1_: Mass of the empty crucible with the lid
m_2_: Mass of the crucible with lid and the sample before heating
m_3_: Mass of the crucible with lid and the sample after heating

### 2.5. Statistical Treatment

#### 2.5.1. Measured Variables Error

Following [20], the batch to be sampled can be considered as a zero dimensional object. Using the sampling procedure described above, a sample of the total batch was chosen. Assuming that the sampling procedure is correct, the sampling error, SE, can be expressed as the sum of two components: the fundamental error, FE, and the segregation and grouping error, SGE. Moreover, as these two errors are independent, the following relationship between their variances holds:

(4) $$\sigma^{2}(SE)=\sigma^{2}(FE)+\sigma^{2}(SGE)$$

The fundamental error is related to the constitutional heterogeneity, is never zero and is the minimum sampling error that can be made. The variance in the fundamental error can be expressed as:

(5) $$\sigma^{2}(FE)=\;\left(\frac{1}{M_{m}}-\frac{1}{M_{L}}\right)\;{HI}_{L}$$

where HI_L_ is the heterogeneity invariant given by:

(6) $${HI}_{L}=\frac{1}{N_{F}}{\sum_{i=1}^{N_{F}}\left(\frac{a_{i}-a_{L}}{a_{L}}\right)}^{2}\;M_{i}$$

In view of the above expressions, it is easy to deduce that the variance of the fundamental error is zero if, and only if, the sample is the whole batch, *M_m_* = *M_L_*, or the material is completely homogeneous, *a_i_* = *a_L_*, *i =* 1,2, …, *N_F_*.

The segregation and grouping error is related to distributional heterogeneity. The variance in the grouping and segregation error, σ^2^ (*SGE*), cannot be calculated, but as the relationship: 0 ≤ σ^2^ (*SGE*) ≤ σ^2^ (*FE*) is verified, then the following relationship can be easily deduced:

(7) $$\sigma^{2}(FE)\;\leq \;\sigma^{2}(SE)\;\leq \;\;2\sigma^{2}(FE)$$

Assuming that the sampling error follows a normal distribution, *i.e., SE ∼ N* (0,σ(*SE*)), we can ensure with a confidence level of 95% that:

(8) $$|SE|\;\leq \;1.96\sqrt{2}\;\sigma (FE)\;=\;1.96\sqrt{2}\sqrt{\left(\frac{1}{M_{m}}-\frac{1}{M_{L}}\right)\;{HI}_{L}}$$

Finally, assuming that M_m_ <<<M_L_, it is easy to get to:

(9) $$|SE|\;\leq \;1.96\;\sqrt{\frac{2{HI}_{L}}{M_{m}}}$$

Using the latest inequality some useful bounds, with a confidence level of 95%, can be inferred for the sampling error and the mass of the sample:

1. If the mass of the sample is constant, the sampling error has an upper bound of a maximum sampling error given by:
  (10) $$|SE|\;\leq \;{SE}_{\text{max}}=\sqrt{7.68\frac{{HI}_{L}}{M_{m}}}$$
2. If a maximum sampling error is set then the mass of the sample should be:
  (11) $$M_{m}\;\geq \;7.68\frac{{HI}_{L}}{{SE}_{\text{max}}^{2}}$$

More details about these results can be seen in [27].

#### 2.5.2. Calculated Variable Error

Since the percentage of fixed carbon can be obtained directly from the other properties of the materials (*FC = 100-M-V-A*), a study of the propagation of error can be carried out. Given the linear relationship between fixed carbon and the other properties and since there is no correlation between moisture, volatiles content and ash content, a straightforward application of the simple propagation of error formula leads to an approximation of the maximum sampling error for fixed carbon:

(12) $${SE}_{1}(FC)=\sqrt{{\left({SE}_{\text{max}}(M)\right)}^{2}+{\left({SE}_{\text{max}}(V)\right)}^{2}+{\left({SE}_{\text{max}}(A)\right)}^{2}}$$

Here *SE_max_*(*M*), *SE_max_*(*V*) and *SE_max_*(*A*) stand for the maximum sampling error for moisture, volatiles and ash respectively.

On the other hand, using the linear relationship between the properties, some simple arithmetic can be used to get a new expression for the heterogeneity invariant of fixed carbon:

(13) $$\begin{array}{c} {HI}_{L}(FC)=\frac{1}{N_{F}}\sum_{i=1}^{N_{F}}{\left(\frac{{FC}_{i}-\bar{FC}}{\bar{FC}}\right)}^{2}\;=\;\frac{1}{{\bar{FC}}^{2}}\frac{1}{N_{F}}\sum_{i=1}^{N_{F}}{\left(M-\bar{M}+V_{i}-\bar{V}+A-\bar{A}\right)}^{2}=\\ =\frac{1}{{\bar{\text{FC}}}^{2}}\frac{1}{{\text{N}}_{\text{F}}}\sum_{i=1}^{{\text{N}}_{\text{F}}}\left[{\left({\text{M}}_{\text{i}}-\bar{\text{M}}\right)}^{2}+{\left({\text{V}}_{\text{i}}-\bar{\text{V}}\right)}^{2}+{\left({\text{A}}_{\text{i}}-\bar{\text{A}}\right)}^{2}+2\left({\text{M}}_{\text{i}}-\bar{\text{M}}\right)\left({\text{V}}_{\text{i}}-\bar{\text{V}}\right)+2\left({\text{M}}_{\text{i}}-\bar{\text{M}}\right)\left({\text{A}}_{\text{i}}-\bar{\text{A}}\right)+2\left({\text{V}}_{\text{i}}-\bar{\text{V}}\right)\left({\text{A}}_{\text{i}}-\bar{\text{A}}\right)\right] \end{array}$$

where HI_L_ stands for the heterogeneity invariant and *M̄*, *V̄*, *Ā*, and *FC* are the sample means of moisture, volatiles, ash and fixed carbon, respectively. On the other hand, no significant correlation is found between the properties of the different materials. Due to this lack of correlation some of the terms included in the latest equation are zero. Then:

(14) $${HI}_{L}(FC)=\frac{1}{{\bar{FC}}^{2}}\frac{1}{N_{F}}\sum_{i=1}^{N_{F}}\left[{\left(M_{i}-\bar{M}\right)}^{2}+{\left(V_{i}-\bar{V}\right)}^{2}+{\left(A_{i}-\bar{A}\right)}^{2}\right]$$

By applying expression 6 it is easy to show the following relationship between the heterogeneity invariant of the properties:

(15) $${HI}_{L}(FC)=\frac{1}{{\bar{FC}}^{2}}\left({\bar{M}}^{2}{HI}_{L}\left(M\right)+{\bar{V}}^{2}{HI}_{L}(V)+{\bar{A}}^{2}{HI}_{L}(A)\right)$$

Finally, using expression 10 a second approximation for the sampling error can be obtained:

(16) $${SE}_{2}(FC)=\sqrt{\frac{7.68}{M_{m}}\times \frac{{\bar{M}}^{2}{HI}_{L}(M)+{\bar{V}}^{2}{HI}_{L}(V)+{\bar{A}}^{2}{HI}_{L}\left(A\right)}{{\left(100-\bar{M}-\bar{V}-\bar{A}\right)}^{2}}}$$

## 3. Results and Discussion

The results of the experimental tests are compiled in this section. Table 2 shows the figures for moisture, ash and volatile contents for all the samples of biofuel used in this study. The variance of the analysis is shown next to the mean levels.

**Figure 3.**
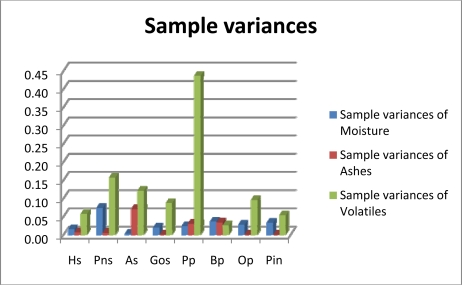
Moisture, Ash and Volatiles variances.

A look at the variance in the properties for all the samples in Tables 2 and 3 reveals that fixed carbon variance levels are lower than the maximum variances associated with every other fuel except brassica. The moisture levels observed in hazelnut shell, pine nut, almond and olive stone were quite similar. These materials were received in big-bags. All the pellets packed in sacks also had similar moisture contents with the exception of brassica pellets, which had a higher percentage. The high ash content in brassica pellets is significant. The results also show a high level of consistency in the average levels of ashs in hazelnut shells (1.10%), pine nut (1.32%) and almond (1.17%). The lowest ash content was found in oak, olive stones and pine pellets. These levels make these pellets best suited for burning in boilers. Poplar pellets were found to have a high ash content. Figure 3 illustrates the variances in moisture, ash and volatiles for the materials studied.

It can be concluded from an analysis of the variances in moisture and ash obtained for the different materials that the sampling methodology is somewhat dependant on the nature of the biofuel. The properties of each material need to be taken into account if adequate accuracy and reliability are to be achieved. For example, materials such as olive stones, pine pellets and oak pellets have a very low variance for ash. On the other hand, their moisture contents vary significantly. The results for almond shell and pine nut shell are surprising, with contrasting variance levels for the two properties. This calls for different sampling plans if the same accuracy and reliability levels are to be achieved in the results. As the moisture in each material depends on its inherent characteristics and on external actions to which it is subjected, greater variances were expected than for ash. This hypothesis was confirmed in only five of the materials.

A correlation analysis of the properties of the biofuels was conducted and no statistically significant correlations were found. This means that the figures for one property, say moisture, cannot be explained by the figures for the others, *i.e.*, ash and volatiles, since there is no linear relationship between them. All three need to be studied separately and no previous analysis of one property can be used to infer the levels of the others.

### 3.1. Error Propagation and Generation of Sampling Maps

For the calculations shown below, the fragment is assumed to be a dimensionless unit of mass M_i_ = 1, so that the mass sample is represented as N_F_ sampling units. To determine the accuracy of the approximations deduced in the previous section, and since the exact sampling error is impossible to determine, the figures for of *SE*_1_ (*FC*) and *SE*_2_ (*FC*) were compared to those obtained using equation 10, shown in Table 4 and also in Table 14 for a different sample size, denoted by *SE_max_*(*FC*). The latter is the best approximation of the actual sampling error that can be achieved, and close coincidence with this figure should indicate a good approximation of the actual sampling error.

Table 4 shows the values of *SE_max_*(*FC*) and *SE*_1_ (*FC*). The correlation coefficient between the second and third columns of the table is −3.64E-02 with a p-value of 0.93, which means a lack of correlation between the maximum sampling error and this first approximation.

Values of *SE*_2_ (*FC*), assuming a sample size of one unit, are included in the fourth column of Table 4. The correlation coefficients between the different approximations for the sampling error, along with their corresponding p-values, are shown in Table 5. The correlation coefficient between the second and fourth columns of Table 4 is 9.77·10^−1^, with a negligible p-value, which indicates a significant correlation between the maximum sampling error and this second approximation. Notice that to calculate *SE*_1_ (*FC*) and *SE*_2_ (*FC*) only the moisture, volatiles and ashes of the materials need to be known, which means that both approximations can be derived from previous studies of the materials. Nevertheless the second approximation seems to work better than the first since additional studies of the data are used.

By applying the statistical treatment described above to the sample data, the values of HI_L_ shown in Table 6 are obtained. With these figures, it can be deduced that the maximum sampling error for a fixed sample mass and the mass of a minimum sample size have a fixed sampling error. These results are given in Tables 7 and 8 for moisture data. Tables 9 and 10 show the data for ash, Tables 11 and 12 for volatiles and Tables 13 and 14 for fixed carbon.

Given a maximum acceptable sample error, with these tables it is possible to establish a minimum sample size for determining levels of moisture, ash, volatiles and fixed carbon, respectively with a confidence level of 95%, (Tables 7, 9, 11 and 13). Alternatively, for a predetermined sample size, it is possible to determine the maximum permissible error for a sample size, which is necessary for determining moisture, ash, volatiles and fixed carbon levels respectively (Tables 8, 10, 12 and 14). The sampling errors are correlated to some extent with the results for variance in Table 2. Those materials with large sample variances will, in general, have higher sampling errors.

## 4. Conclusions

When the representative properties of heterogeneous biomass substances are determined in batches, a sampling methodology must be established for each property. This paper introduces a new sampling process and provides a statistical analysis, defining a sampling error or level of uncertainty associated with the properties measured. This is crucial for learning the subsequent propagation of error in future calculations with the set property level. The new methodology is validated by means a prompt analysis variance analysis.

Although they are heterogeneous materials, the biofuels studied here show reasonable limits. In other words, despite the heterogeneity of the fuel itself a well-planned campaign of samples can extrapolate the properties of samples from the entire batch with a controlled, analyzed, quantified level of uncertainty.

The paper also shows that sample variance cannot accurately quantify error levels. The statistical uncertainty associated with this property needs to be determined for errors to be quantified precisely. The sampling procedure and statistical determination techniques can be extrapolated to any other solid material in granular form with approximately homogeneous sizes.

Sampling errors are significantly correlated with sample variances. Thus, materials with high levels of sample variance will, in general, have higher sampling errors. In the case of moisture, the correlation coefficient between the sampling error and the sample variance is 0.69. Correlation increases to 0.79 for ash, 0.96 for volatiles and up to 0.98 for fixed carbon. It can thus be deduced that sample variance is more of a qualitative than a quantitative indicator of sampling errors but that in no case can it be estimated. Perfect correlation (1.00) is achieved between the coefficient of variation (ratio of the standard deviation to the mean) and the sampling error. This result applies to all materials and is a consequence of the mathematical expression of the heterogeneity invariant and, therefore, of the expression used to obtain the maximum sampling error.

The correlation coefficients between the maximum sampling errors obtained for the different properties were calculated. Only the correlation between moisture and ashes seemed to be significant, with a coefficient of −0.76. Further study of the data leads to the conclusion that this figure is a consequence of the atypical behaviour of the almond shell. When this single observation is omitted the coefficient changes to −0.48. In view of these coefficients, the maximum sampling error of a given property should not be approximated from the maximum sampling errors of the other properties. This might explain the lack of correlation between *SE_max_*(*CF*) and aproximation *SE*_1_(*CF*) given in the previous section.

## Figures and Tables

**Table 1.** Rounded-off average weight of the samples.

	**Weight of Laboratory Sample**		
**Material**	**Moisture kg**	**Ash kg**	**Volatiles kg**
Hazelnut shell (Hs)	21.7 × 10^−3^	8.5 × 10^−3^	23.1× 10^−3^
Pine nut shell (Pns)	17.9 × 10^−3^	6.8 × 10^−3^	6.9 × 10^−3^
Almond shell (As)	23.9 × 10^−3^	9.2 × 10^−3^	9.8 × 10^−3^
Ground olive stone (Gos)	18.1 × 10^−3^	7.8 × 10^−3^	7.8 × 10^−3^
Poplar pellets (Pp)	14.0 × 10^−3^	8.2 × 10^−3^	6.2 × 10^−3^
Brassica pellets (Bp)	13.9 × 10^−3^	3.1 × 10^−3^	6.3 × 10^−3^
Oak pellets (Op)	21.7 × 10^−3^	8.8 × 10^−3^	20.2 × 10^−3^
Pine pellets (Pin)	19.0 × 10^−3^	10.1 × 10^−3^	17.8 × 10^−3^

**Table 2.** Levels of moisture, ash and volatile content in the biofuels tested in %.

**Material**	**Propety**	**Sample 1**	**Sample 2**	**Sample 3**	**Sample 4**	**Sample 5**	**Sample 6**	**Sample 7**	**Sample 8**	**Sample 9**	**Sample 10**	**Mean**	**S^2^**
	Moist	12.264	12.055	11.961	12.189	11.876	12.075	11.998	12.038	11.934		12.043	0.01
Hs	Ash	1.037	0.878	0.937	0.956	0.897	0.993	1.002	1.149	0.929		0.975	0.00
	Volati	64.418	64.568	64.960	64.452	64.932	64.556	64.937	64.572	64.993		64.710	0.05
	Moist	12.063	12.102	12.184	12.419	12.208	12.695	12.701	12.683	12.154		12.357	0.07
Pns	Ash	1.258	1.264	1.210	1.119	1.171	1.094	1.103	1.091	1.093		1.156	0.00
	Volati	67.149	67.334	67.043	66.889	66.555	66.030	66.685	66.599	66.493		66.753	0.15
	Moist	12.621	12.632	12.562	12.628	12.533	12.589	12.643	12.643	12.498		12.594	0.00
As	Ash	0.858	1.078	1.560	1.248	0.851	0.896	1.181	0.802	0.749		1.025	0.07
	Volati	68.731	68.024	67.849	68.611	68.577	68.897	68.620	68.766	68.523		68.511	0.12
	Moist	12.629	12.658	12.812	12.394	12.416	12.623	12.698	12.764	12.595		12.621	0.01
Gos	Ash	0.477	0.501	0.485	0.451	0.460	0.465	0.502	0.475	0.517		0.481	0.00
	Volati	69.656	70.206	69.345	69.783	69.814	70.206	69.503	69.761	69.547		69.758	0.08
	Moist	8.025	8.044	7.701	7.816	8.016						7.920	0.02
Pp	Ash	2.507	2.809	2.957	2.633	2.796						2.740	0.03
	Volati	73.479	73.744	74.902	74.639	73.548						74.062	0.43
	Moist	10.301	9.907	10.344	10.005	10.070						10.125	0.03
Bp	Ash	8.903	8.807	8.430	8.828	8.764						8.746	0.03
	Volati	66.593	66.768	66.461	66.850	66.790						66.692	0.02
	Moist	7.568	7.515	7.479	7.742	7.595	7.549	7.182	7.302	7.675	7.453	7.506	0.02
Op	Ash	0.704	0.722	0.711	0.707	0.705	0.746	0.753	0.741	0.735	0.696	0.722	0.00
	Volati	73.239	72.985	73.812	72.723	72.968	72.987	73.310	73.259	----------	73.052	73.148	0.09
	Moist	7.350	7.209	7.605	7.449	7.288	7.366	7.063	7.695	7.411	7.348	7.378	0.03
Pin	Ash	0.482	0.485	0.439	0.485	0.492	0.485	0.480	0.455	0.491	0.485	0.478	0.00
	Volati	74.757	74.595	74.908	74.566	74.708	74.748	75.081	74.475	74.415	74.320	74.657	0.05

It was not possible to calculate the fixed carbon content of the oak pellet in sample nine (Op) because the sample became corrupted during the process to determine the volatiles content. This lack of information was of course taken into account in the calculations. Table 3 shows the fixed carbon levels obtained for each sample, the average level the variance.

**Table 3.** Calculated fixed carbon in wet basis.

**Material**	**Sample 1**	**Sample 2**	**Sample 3**	**Sample 4**	**Sample 5**	**Sample 6**	**Sample 7**	**Sample 8**	**Sample 9**	**Sample 10**	**Mean**	**S^2^**
Hs	22.281	22.500	22.142	22.402	22.296	22.376	22.063	22.241	22.144		22.272	0.0197
Pns	19.531	19.300	19.562	19.573	20.066	20.181	19.511	19.628	20.260		19.735	0.1166
As	17.789	18.266	18.029	17.512	18.039	17.618	17.556	17.789	18.230		17.870	0.0805
Gos	17.238	16.635	17.358	17.372	17.310	16.706	17.297	17.001	17.341		17.140	0.0834
Pp	15.990	15.403	14.440	14.912	15.639						15.277	0.3723
Bp	14.203	14.519	14.765	14.317	14.375						14.436	0.0468
Op	18.489	18.778	17.998	18.828	18.732	18.719	18.755	18.698	---------------	18.800	18.644	0.0683
Pin	17.411	17.710	17.047	17.501	17.512	17.401	17.377	17.375	17.683	17.848	17.487	0.0500

**Table 4.** Maximum sampling errors and two approximations for fixed carbon, assuming a sample size of one unit.

	***SE_max_*(*FC*)**	***SE*_1_(*FC*)**	***SE*_2_(*FC*)**
Hs	1.65E-02	2.23E-01	3.30E-02
Pns	4.52E-02	1.74E-01	6.42E-02
As	4.15E-02	6.77E-01	6.44E-02
Gos	4.40E-02	1.21E-01	4.99E-02
Pp	9.90E-02	1.66E-01	1.14E-01
Bp	3.71E-02	7.01E-02	5.31E-02
Op	3.66E-02	9.48E-02	4.93E-02
Pin	3.36E-02	1.14E-01	4.42E-02

**Table 5.** Pearson correlation coefficients between maximum sampling error and two approximations for fixed carbon, assuming a sample size of one unit. P-values in brackets.

	***SE_max_*(*FC*)**	***SE*_1_(*FC*)**	***SE*_2_(*FC*)**
*SE_max_*(*FC*)	1		
*SE*_1_(*FC*)	−3.64E-02 (0.93)	1	
*SE*_2_(*FC*)	9.77E-01 (0.000)	1.11E-01 (0.79)	1

**Table 6.** Values for the intrinsic heterogeneity of moisture and ash concentrations observed in different biomass materials.

	**HI_L_**			
	**Moisture**	**Ash**	**Volatiles**	**Fixed Carbon**
Hs	9.21E-05	6.38E-03	1.22E-05	3.54E-05
Pns	4.28E-04	3.46E-03	3.13E-05	2.66E-04
As	1.55E-05	5.97E-02	2.28E-05	2.24E-04
Gos	1.11E-04	1.79E-03	1.59E-05	2.52E-04
Pp	3.02E-04	3.21E-03	6.36E-05	1.28E-03
Bp	2.81E-04	3.53E-04	4.67E-06	1.80E-04
Op	4.40E-04	7.14E-04	1.58E-05	1.75E-04
Pin	5.44E-04	1.13E-03	8.61E-06	1.47E-04

**Table 7.** Moisture. Minimum sample mass, expressed as N_m_ sampling units, sampling error for a determined maximum sampling error.

		**Minimum sample size for a determined sampling error**							
		Hs	Pns	As	Gos	Pp	Bp	Op	Pin
	
	**HI_L_**	9.21E-05	4.28E-04	1.55E-05	1.11E-04	3.02E-04	2.81E-04	4.40E-04	5.44E-04

Maximum error	0.001	7.08E+02	3.29E+03	1.19E+02	8.50E+02	2.32E+03	2.16E+03	3.38E+03	4.18E+03

	0.005	2.83E+01	1.31E+02	4.76E+00	3.40E+01	9.27E+01	8.64E+01	1.35E+02	1.67E+02

	0.01	7.08E+00	3.29E+01	1.19E+00	8.50E+00	2.32E+01	2.16E+01	3.38E+01	4.18E+01

	0.05	2.83E-01	1.31E+00	4.76E-02	3.40E-01	9.27E-01	8.64E-01	1.35E+00	1.67E+00

**Table 8.** Moisture. Maximum sampling error for a sample mass, expressed as N_m_ sampling units, fixed.

		**Maximum error for the sample size**							
		Hs	Pns	As	Gos	Pp	Bp	Op	Pin
	
	**HI_L_**	9.21E-05	4.28E-04	1.55E-05	1.11E-04	3.02E-04	2.81E-04	4.40E-04	5.44E-04

Sample size	1	2.66E-02	5.73E-02	1.09E-02	2.92E-02	4.81E-02	4.65E-02	5.82E-02	6.47E-02
	10	8.41E-03	1.81E-02	3.45E-03	9.22E-03	1.52E-02	1.47E-02	1.84E-02	2.04E-02
	100	2.66E-03	5.73E-03	1.09E-03	2.92E-03	4.81E-03	4.65E-03	5.82E-03	6.47E-03
	200	1.88E-03	4.05E-03	7.71E-04	2.06E-03	3.40E-03	3.29E-03	4.11E-03	4.57E-03

**Table 9.** Ash. Minimum sample mass, expressed as N_m_ sampling units, sampling error for a determined maximum sampling error.

		**Minimum sample size for a determined sampling error**							
		Hs	Pns	As	Gos	Pp	Bp	Op	Pin
	
	**HI_L_**	6.38E-03	3.46E-03	5.97E-02	1.79E-03	3.21E-03	3.53E-04	7.14E-04	1.13E-03

Maximum error	0.001	4.90E+04	2.66E+04	4.58E+05	1.38E+04	2.47E+04	2.71E+03	5.49E+03	8.66E+03

	0.005	1.96E+03	1.06E+03	1.83E+04	5.52E+02	9.88E+02	1.08E+02	2.19E+02	3.47E+02

	0.01	4.90E+02	2.66E+02	4.58E+03	1.38E+02	2.47E+02	2.71E+01	5.49E+01	8.66E+01

	0.05	1.96E+01	1.06E+01	1.83E+02	5.52E+00	9.88E+00	1.08E+00	2.19E+00	3.47E+00

**Table 10.** Ashes. Maximum sampling error for a sample mass, expressed as N_m_ sampling units, fixed.

		**Maximum error for the sample size**							
		Hs	Pns	As	Gos	Pp	Bp	Op	Pin
	
	**HI_L_**	6.38E-03	3.46E-03	5.97E-02	1.79E-03	3.21E-03	3.53E-04	7.14E-04	1.13E-03

Sample size	1	2.21E-01	1.63E-01	6.77E-01	1.17E-01	1.57E-01	5.21E-02	7.41E-02	9.31E-02
	10	7.00E-02	5.16E-02	2.14E-01	3.71E-02	4.97E-02	1.65E-02	2.34E-02	2.94E-02
	100	2.21E-02	1.63E-02	6.77E-02	1.17E-02	1.57E-02	5.21E-03	7.41E-03	9.31E-03
	200	1.57E-02	1.15E-02	4.79E-02	8.30E-03	1.11E-02	3.68E-03	5.24E-03	6.58E-03

**Table 11.** Volatiles. Minimum sample mass, expressed as N_m_ sampling units, sampling error for a determined maximum sampling error.

		**Minimum sample size for a determined sampling error**							
		Hs	Pns	As	Gos	Pp	Bp	Op	Pin
	
	**HI_L_**	1.22E-05	3.13E-05	2.28E-05	1.59E-05	6.36E-05	4.67E-06	1.58E-05	8.61E-06

Maximum error	0.001	9.34E+01	2.40E+02	1.76E+02	1.22E+02	4.89E+02	3.59E+01	1.22E+02	6.62E+01

	0.005	3.74E+00	9.61E+00	7.02E+00	4.88E+00	1.96E+01	1.44E+00	4.87E+00	2.65E+00

	0.01	9.34E-01	2.40E+00	1.76E+00	1.22E+00	4.89E+00	3.59E-01	1.22E+00	6.62E-01

	0.05	3.74E-02	9.61E-02	7.02E-02	4.88E-02	1.96E-01	1.44E-02	4.87E-02	2.65E-02

**Table 12.** Volatiles. Maximum sampling error for a sample mass, expressed as N_m_ sampling units, fixed.

		**Maximum error for the sample size**							
		Hs	Pns	As	Gos	Pp	Bp	Op	Pin
	
	**HI_L_**	1.22E-05	3.13E-05	2.28E-05	1.59E-05	6.36E-05	4.67E-06	1.58E-05	8.61E-06

Sample size	1	9.66E-03	1.55E-02	1.32E-02	1.10E-02	2.21E-02	5.99E-03	1.10E-02	8.13E-03
	10	3.06E-03	4.90E-03	4.19E-03	3.49E-03	6.99E-03	1.89E-03	3.49E-03	2.57E-03
	100	9.66E-04	1.55E-03	1.32E-03	1.10E-03	2.21E-03	5.99E-04	1.10E-03	8.13E-04
	200	6.83E-04	1.10E-03	9.37E-04	7.81E-04	1.56E-03	4.24E-04	7.80E-04	5.75E-04

**Table 13.** Fixed carbon. Minimum sample mass, expressed as N_m_ sampling units, sampling error for a determined maximum sampling error.

		**Minimum sample size for a determined sampling error**							
		Hs	Pns	As	Gos	Pp	Bp	Op	Pin
	
	**HI_L_**	3.54E-05	2.66E-04	2.24E-04	2.52E-04	1.28E-03	1.80E-04	1.75E-04	1.47E-04

Maximum error	0.001	2.72E+02	2.04E+03	1.72E+03	1.94E+03	9.81E+03	1.38E+03	1.34E+03	1.13E+03

	0.005	1.09E+01	8.18E+01	6.88E+01	7.75E+01	3.92E+02	5.52E+01	5.37E+01	4.53E+01

	0.01	2.72E+00	2.04E+01	1.72E+01	1.94E+01	9.81E+01	1.38E+01	1.34E+01	1.13E+01

	0.05	1.09E-01	8.18E-01	6.88E-01	7.75E-01	3.92E+00	5.52E-01	5.37E-01	4.53E-01

**Table 14.** Fixed carbon. Maximum sampling error for sample mass, expressed as N_m_ sampling units, fixed.

		**Maximum error for the sample size**							
		Hs	Pns	As	Gos	Pp	Bp	Op	Pin
	
	**HI_L_**	3.54E-05	2.66E-04	2.24E-04	2.52E-04	1.28E-03	1.80E-04	1.75E-04	1.47E-04

Sample size	1	1.65E-02	4.52E-02	4.15E-02	4.40E-02	9.90E-02	3.71E-02	3.66E-02	3.36E-02
	10	5.21E-03	1.43E-02	1.31E-02	1.39E-02	3.13E-02	1.17E-02	1.16E-02	1.06E-02
	100	1.65E-03	4.52E-03	4.15E-03	4.40E-03	9.90E-03	3.71E-03	3.66E-03	3.36E-03
	200	1.17E-03	3.20E-03	2.93E-03	3.11E-03	7.00E-03	2.63E-03	2.59E-03	2.38E-03
